# Clinical and Pain-Related Characteristics of Idiopathic First Bite Syndrome Induced by Taste in Japanese Patients without Diabetes: A Retrospective Study of Five Cases

**DOI:** 10.1155/2021/6674102

**Published:** 2021-02-13

**Authors:** Masatoshi Chiba, Hiroaki Hirotani, Tetsu Takahashi

**Affiliations:** ^1^Division of Oral and Maxillofacial Surgery, Department of Disease Management Dentistry, Tohoku University Graduate School of Dentistry, 4-1 Seiryo-machi, Aoba-ku, Sendai 980-8575, Miyagi, Japan; ^2^Dentistry and Oral Surgery, Oosaki Citizen Hospital, 3-8-1 Honami, Furukawa, Oosaki 989-6183, Miyagi, Japan

## Abstract

**Objective:**

First bite syndrome (FBS) is a condition in which the first bite of each meal causes parotid pain. Etiologies of FBS include prior surgery of the upper cervical region and, rarely, head and neck tumors. Idiopathic FBS rarely presents in patients without a history of surgery or evidence of an underlying tumor. Idiopathic FBS may be categorized into two subtypes: that in patients with diabetes and that in patients without diabetes. Idiopathic FBS in patients without diabetes may be overlooked or misdiagnosed because the condition has been described only in a few case reports. We aimed to identify the clinical and pain-related characteristics of idiopathic FBS in patients without diabetes.

**Methods:**

We retrospectively analyzed the clinical data of five patients without diabetes who were diagnosed with idiopathic FBS in our department between January 2010 and December 2016.

**Results:**

Four of the five patients were female, and the overall median age was 52 years (range: 13–61). All patients immediately experienced parotid pain upon tasting food without chewing. Addition of an acidic solution to the ipsilateral posterior third of the tongue evoked parotid pain. The median degree of pain intensity and interference with eating due to pain was 9 (range: 3–10) and 9 (range: 5–10) on a numerical rating scale of 0–10, respectively. Idiopathic FBS was bilateral in two patients. Two patients had tenderness on mild pressure over the affected parotid region. Two patients presented with ipsilateral idiopathic Horner's syndrome.

**Conclusions:**

Our findings indicate that the characteristics of idiopathic FBS in patients without diabetes are largely consistent with those previously reported in postoperative FBS, supporting the notion that idiopathic FBS is a subtype of FBS. Thus, it is necessary to consider idiopathic FBS during the evaluation of facial pain triggered at the beginning of a meal.

## 1. Introduction

First bite syndrome (FBS) is a relatively rare clinical condition in which the first bite of each meal causes severe pain or cramping that gradually subsides with subsequent mastication [1, 2]. Patients may avoid eating because of the severity of FBS pain. Potential treatments for relieving FBS pain include dietary modifications [1], pharmacological interventions [3–6], and botulinum toxin injection [6, 7]. However, owing to the limited number of cases that have addressed the management of FBS, there is no established therapy for this condition.

The etiology and pathogenesis of FBS remain to be elucidated. FBS mainly occurs after upper neck surgery; therefore, most studies have focused on the association between FBS development and operative procedures [1, 2, 4–6, 8, 9]. The strongest independent factors for FBS are parapharyngeal space dissection, resection of only the deep lobe of the parotid gland, and sympathetic chain sacrifice [4]. Collectively, these factors suggest that the loss of sympathetic innervation to the parotid gland and consequent denervation hypersensitivity of the parotid myoepithelial cells cause FBS pain [1, 2, 4–6, 8–10].

FBS in the absence of prior surgical intervention is rare; therefore, it has mainly been described only in case reports. The reports suggest that FBS is associated with tumors, namely, malignant tumors arising from the parapharyngeal space [11, 12], parotid gland [13], or submandibular gland [14] and benign tumors of the temporal bone [15]. FBS may rarely occur in patients without a history of surgery of the upper cervical region or evidence of an underlying tumor. However, diabetic neuropathy is another potential cause of FBS. Recent studies have documented parotid pain compatible with FBS in patients with diabetes [16, 17].

In rare cases, FBS may be idiopathic [18, 19]; therefore, it may be overlooked or easily misdiagnosed during the evaluation of facial pain. As such, it poses a diagnostic challenge for even the most experienced pain clinicians. Furthermore, the characteristics of idiopathic FBS have not been thoroughly investigated. Therefore, the aim of this study was to identify the clinical and pain-related characteristics of idiopathic FBS in patients without diabetes.

## 2. Patients and Methods

### 2.1. Patients

We included all patients who were diagnosed with FBS in the Department of Oral and Maxillofacial Surgery at Tohoku University Hospital over a 6-year period between January 2010 and December 2016. The study was performed in accordance with the standards outlined in the Declaration of Helsinki and was approved by our Institutional Review Board (2016-3-25). Written, informed consent for participation in this study was not obtained from the patients due to the retrospective nature of the study. However, we made a public announcement on our institution's Internet homepage as requested by the Institutional Review Board. We diagnosed patients with idiopathic FBS if they met the following four criteria: (1) pain in the parotid region occurring on the first few bites of each meal, which improved with subsequent mastication; (2) no evidence of parotid gland, masticatory muscle, temporomandibular joint, or odontogenic/neurologic pathology as the cause of facial pain; (3) no history of surgery involving the upper neck region; and (4) no detectable tumors arising from the head or neck on magnetic resonance imaging (MRI) [16]. We excluded patients with diabetes and used the term idiopathic parotid pain to describe idiopathic FBS in those with diabetes [16].

### 2.2. Data Collection

Clinical data were obtained from the medical records, including the examination protocols and questionnaires utilized in our orofacial pain clinic. The following factors were analyzed: patient characteristics; clinical, laboratory, and imaging findings; and clinical course of idiopathic FBS. Patient characteristics included age, sex, medical history, duration of idiopathic FBS, and diagnosis and treatment before referral. The anatomical location/side of idiopathic FBS pain and the sites of pain on the face and neck upon palpation were recorded. Sensory abnormalities of the oral mucosa and facial skin on touching with a cotton swab were also recorded along with any autonomic symptoms.

All imaging findings were reevaluated. These included panoramic radiography, 1.5-T MRI of the head and neck (T1-weighted, T2-weighted, fat-suppressed T2-weighted, and fast imaging employing steady-state acquisition centered on the trigeminal nerve for facial pain), 1.5-T MRI of the temporomandibular joints, and salivary gland scintigraphy (time-activity curves, accumulation, and rate of excretion in the parotid glands) [20].

We also reviewed the following: (1) whether the application of an acidic solution with a cotton swab to the tongue elicited parotid pain; (2) whether chewing a cotton swab or gauze triggered pain; (3) severity of pain and interference with eating over a 7-day period; and (4) the duration and quality of idiopathic FBS pain. Pain and interference with eating were rated using an 11-point numerical scale. For pain, the scale ranged from 0 (“no pain”) to 10 (“worst pain imaginable”); for interference with eating due to pain, the scale ranged from 0 (“does not interfere”) to 10 (“completely interferes”). Improvements in idiopathic FBS were documented when patients experienced a decrease in pain intensity following therapy.

## 3. Results

### 3.1. Characteristics of Patients with Idiopathic FBS

Of the 18 cases of FBS that were reviewed, 13 were excluded because of a diagnosis of diabetes (*n* = 11), temporal tumors (*n* = 1), or cervical cysts (*n* = 1). Of the remaining five patients, four were women, and the overall median age was 52 years (range: 13–61) (Table 1). All five patients were referred from other hospitals or dental offices. The median duration of idiopathic FBS was 36 months (range: 1.5 months–10 years); none of the patients had experienced remission (Table 1). Patient 3 was initially misdiagnosed with trigeminal neuralgia by a neurosurgeon at another hospital. Initial management with carbamazepine and pregabalin did not alleviate the pain. The patient then underwent microvascular decompression; however, facial pain persisted postoperatively. Repeat microvascular decompression was considered, but the patient declined the procedure. The remaining four patients were initially misdiagnosed with temporomandibular disorders by their dentists; splints were applied in Patients 1, 2, and 5, which did not relieve the pain. Patient 1 received medical treatment for orthostatic hypotension and abdominal pain followed by the onset of idiopathic FBS.

### 3.2. Clinical Characteristics of Idiopathic FBS

Idiopathic FBS was bilateral in two patients and unilateral in three patients (Table 2). The pain was confined to the parotid region in four patients and additionally spread along the mandible in one patient (Patient 2). Two patients had tenderness over the affected parotid region upon mild pressure. None of the following were observed in any of the patients: (1) swelling in the parotid region; (2) pain upon manipulation of the temporomandibular joints; and (3) sensory deficits or dysesthesia of the orofacial region on neurological examination.

Patient 5 had developed right-sided Horner's syndrome a few years before the onset of idiopathic FBS; therefore, the patient was examined by an ophthalmologist, neurologist, and pulmonologist. MRI and computed tomography of the entire oculosympathetic chain revealed no pathologies in the head, neck, or chest region that might have caused Horner's syndrome. Patient 3 had miosis on the affected side and was diagnosed with idiopathic Horner's syndrome by a neurosurgeon at the previous hospital.

Panoramic radiography was performed to rule out any odontogenic cause or other jaw-related pathologies, and the findings were unremarkable. Initial and follow-up MRI of the head and neck revealed no pathological abnormalities responsible for the parotid pain in any of the patients. MRI of the temporomandibular joints revealed mild anterior disk displacement with reduction in two patients (Patients 2 and 3) (Table 3). Dynamic salivary gland scintigraphy was performed following intravenous injection of 99 m technetium-pertechnetate in four patients. The time-activity curves were normal with a good accumulation of the isotope in the parotid glands. The rate of excretion exceeded 50% following acid loading.

Laboratory blood investigations were performed in all patients. Patient 2 had high titers of antinuclear antibodies and was referred to a rheumatologist who ruled out autoimmune diseases.

### 3.3. Characteristics of Idiopathic FBS Pain

The overall median degree of pain intensity and interference with eating due to pain was 9 (range: 3–10) and 9 (range: 5–10), respectively (Table 4). Parotid pain occurred immediately upon tasting food in the absence of chewing and subsided with subsequent mastication. The pain lasted ≤20 s in all patients, with one patient (Patient 1) initially reporting brief yet severe pain followed by mild pain that lasted for approximately 20 min. Once the first bite pain had subsided, all except Patient 1 resumed eating without pain. However, the pain recurred when chewing was paused for several minutes and restarted at the next meal. In all instances, the features of pain were identical. Tart foods and those with intense flavors caused the most severe pain. The pain was replicated by applying an acidic solution with a cotton swab to the posterior third of the ipsilateral tongue. Chewing a cotton swab or gauze did not evoke parotid pain and neither did speaking or swallowing. No patients experienced postprandial pain of the parotid region.

### 3.4. Clinical Course of Idiopathic FBS

All patients were initially treated with neurotropin (Nippon Zoki Pharmaceutical Co., Ltd., Osaka, Japan), an extract of inflamed cutaneous tissue derived from rabbits inoculated with the vaccinia virus. The median follow-up period in patients was 48 months (range: 3.7–60 months).

Patient 1 was treated with neurotropin (12 NU/day) for 7 months, and the pain intensity of the first bite pain decreased from 10 to 2 on the numerical rating scale. She achieved near-complete resolution of the FBS pain after 6 months of cessation of neurotropin. This patient has been followed up for 32 months without obvious signs of recurrence. Patient 3 was treated with neurotropin (16 NU/day), and after 3 months, the intensity of the pain decreased from 9 to 3 on the numerical rating scale. However, she was lost to follow-up. Patients 2 and 5 were unresponsive to neurotropin (16 NU/day); pregabalin was also ineffective and induced drowsiness. Rubbing the affected parotid region before a meal and ingesting bland foods at the beginning of a meal partially alleviated the first bite pain; whenever these measures were not undertaken, the first bite pain worsened. Patient 4 received neurotropin (16 NU/day) for 3 weeks without effect and did not receive any further treatment because the parotid pain was tolerable. However, the pain gradually subsided over the ensuing 5 months.

## 4. Discussion

This study has documented parotid pain consistent with postoperative FBS in patients with no history of surgery, head and neck tumors, or diabetes. The cause of FBS in our study remained unknown; therefore, they are likely to be idiopathic. In our patients, gustatory stimuli reflexively evoked pain in the parotid region, and the trigger zone was located in the posterior one-third of the ipsilateral tongue. It is important to note that two patients also had ipsilateral Horner's syndrome.

The patients in our study experienced sharp pain at the beginning of a meal, which resolved within 20 s but recurred at the next meal or when chewing was paused for several minutes. The first bite pain was intense enough to discourage chewing, and after its decline, 4/5 patients resumed eating painlessly. Unlike a previous study in which 6/9 patients with idiopathic parotid pain presented with severe postprandial pain [16], our patients had no postprandial pain. In these aspects, idiopathic FBS pain largely mimicked postoperative FBS [4, 5] and, thus, may be a subtype of FBS.

However, none of our patients had a history of surgery involving the upper neck region, or structural or functional abnormalities in the involved parotid glands on clinical examination or imaging that could cause severe pain. To the best of our knowledge, this is the first study to demonstrate the absence of functional parotid abnormalities in patients with FBS using salivary scintigraphy. Tumors in the head and neck rarely cause FBS [11–15], and malignant tumors may only become obvious several months after the onset of FBS [14]. Our patients did not have head and neck tumors as confirmed via initial and follow-up MRI and did not have diabetes. Furthermore, unlike postoperative FBS, which always occurs on the operated side [1, 2, 4–6, 8, 9], 2/5 patients in our study experienced bilateral FBS. Therefore, idiopathic FBS and other forms of FBS may have different etiologies.

Idiopathic FBS pain occurs immediately after tasting liquid or solid foods, particularly tart foods. The relationship between gustatory stimuli and reflex stimulation of the parotid gland was confirmed by the elicitation of parotid pain by the application of an acidic solution to the posterior third of the affected tongue. These findings highlight gustatory stimuli, and not chewing, as the FBS trigger and localize the trigger zone to the posterior third of the tongue. Although not required for the elicitation of pain, chewing worsens the pain. The glossopharyngeal nerve conveys taste sensations from the gustatory receptors on the posterior third of the tongue to the nucleus of the solitary tract. It also provides parasympathetic secretomotor innervation to the parotid gland through the neighboring auriculotemporal nerve [21, 22]. Based on our findings, we believe that idiopathic FBS results from the activation of the gustatory-salivary reflex by the glossopharyngeal afferent/parasympathetic efferent circuit innervating the parotid glands. A previous report implicated the activation of the gustatory-parotid-salivary reflex by the glossopharyngeal nerve in the development of idiopathic parotid pain [16]. Unlike idiopathic FBS, postoperative FBS is largely triggered by chewing [1, 2, 4, 6–10]. Additional triggers of pain in patients with postoperative FBS include salivation, thought of food, and contact with food [2, 5, 23]. Of note, in the report by Gardner and Abdullah [24], a gustatory stimulus was identified as the cause of parotid pain consistent with FBS in patients who had undergone superior cervical ganglionectomy. We believe that reexamination of the triggers in postoperative FBS is necessary along with the identification of the accompanying trigger zone, which is currently unknown.

The pathogenesis by which the expulsion of saliva induces severe parotid pain in the absence of anatomical obstruction of the parotid duct remains unknown in the present study. The parotid gland is amply supplied with sensory fibers from the auriculotemporal nerve. Furthermore, parotid myoepithelial cells are dually innervated by the sympathetic and parasympathetic nerves, with both impulses stimulating myoepithelial cell contraction and consequent salivary flow [21, 22]. In 2/5 patients in our study, the affected parotid region was hypersensitive to mild pressure, perhaps, owing to auriculotemporal neuropathy. The autonomic involvement observed in the two patients with pressure hypersensitivity potentially implicates small fiber neuropathy in idiopathic FBS pain. Posttraumatic gustatory neuralgia can develop after head and neck surgery, and taste stimuli can induce paroxysmal pain owing to the abnormal interactions between the salivary efferent fibers and trigeminal sensory afferent fibers in the injured auriculotemporal nerve [25]. However, our patients had no history of surgeries or injuries that may have damaged the auriculotemporal nerve. Therefore, despite similarities, idiopathic FBS and posttraumatic gustatory neuralgia appear to be distinct entities.

Netterville et al. [1] proposed that postoperative FBS occurs secondary to the loss of sympathetic innervation to the parotid gland, which results in denervation hypersensitivity of the sympathetic receptors that control the myoepithelial cells. Mastication would, thus, induce the parasympathetic release of acetylcholine, which results in hyperintense contraction of the myoepithelial cells and the consequent pain with the first bites. Although this hypothesis has not been proven, most investigators agree with it [2, 8–11, 13, 15, 23]. Postoperative FBS may be accompanied by iatrogenic Horner's syndrome following ablation of the sympathetic chain [1, 2, 4, 5, 8]. In the present study, two patients (Patients 3 and 5) had Horner's syndrome. Patient 5 had no pathologies in the head, neck, or chest region that might have caused Horner's syndrome as determined via clinical and imaging examinations of the entire oculosympathetic chain [26]. Gardner and Abdullah [24] reported that degeneration of the postganglionic sympathetic nerves in the parotid due to superior cervical ganglionectomy precedes parotid pain, which never occurs on the side of preganglionic sympathectomy. Therefore, the presence of ipsilateral Horner syndrome suggests that cervical sympathetic neuropathy accounts for idiopathic FBS in these patients; the cause of sympathetic neuropathy, however, is unknown.

There was no clinical evidence of impaired sympathetic innervation of the parotid glands in the remaining three patients (Patients 1, 2, and 4) in our study. However, cervical sympathetic denervation due to ligation of the external carotid artery or excision of the cervical ganglion does not always induce postoperative FBS [4, 5]. Furthermore, recent reports have described atypical cases of FBS involving parasympathetic denervation secondary to trigeminal nerve radiosurgery [27] and chemotherapy for Hodgkin's lymphoma [28]. The findings of these studies and those of the present study suggest that the loss of sympathetic innervation in the parotid gland is not the only mechanism involved in the pathogenesis of FBS. Further studies are required to elucidate the pathophysiological mechanisms of FBS.

Although our cases involved pain characteristic of FBS, they were initially misdiagnosed as trigeminal neuralgia or temporomandibular disorders, which resulted in inappropriate clinical management. Importantly, idiopathic FBS should not be confused with trigeminal neuralgia as this can lead to unnecessary microvascular decompression. Unlike idiopathic FBS, trigeminal neuralgia is associated with brief, electric shock-like pain evoked by innocuous mechanical stimuli, such as light touching of the face, talking, chewing, and brushing [23, 29]. Neurosurgeons should be aware of and rule out idiopathic FBS before performing microvascular decompression in patients with intractable trigeminal neuralgia. Provocation tests, such as the application of an acidic solution to the tongue, may help distinguish idiopathic FBS from trigeminal neuralgia. In our patients, the passive exercise of the temporomandibular joints did not elicit pain, which enabled us to easily exclude temporomandibular joint disorders as the source of the pain.

Although postoperative FBS pain may decrease in severity with time or disappear spontaneously [4–6, 9], none of our patients experienced remission. Additionally, the severity of pain necessitated treatment. Treatment for idiopathic FBS or idiopathic parotid pain includes the administration of pharmacological agents [16, 17, 19] and injection of botulinum toxin [18]; however, the efficacy of these treatments remains to be determined, and a radical cure for idiopathic FBS is lacking. Similar to patients with idiopathic parotid pain [16], two of our patients responded to neurotropin (Nippon Zoki Pharmaceutical Co., Ltd.). It is widely used in Japan as an analgesic agent for treating neuropathic pain, especially postherpetic neuralgia [30]. Although it is thought to activate the descending pain inhibitory system [30], the pharmacological mechanisms by which it resolves parotid pain in patients with idiopathic FBS are presently unknown. In the two patients in whom pregabalin and neurotropin were ineffective, rubbing the parotid region before a meal and ingesting bland foods at the beginning of a meal partially relieved the pain. In contrast to acidic/intense taste stimuli, which induce severe pain, these remedies may dampen the abrupt increases in salivation at the beginning of a meal and, thus, reduce pain severity.

Our study has some limitations, such as the retrospective, single-center design and a small number of cases. Therefore, our findings may not be generalizable to all patients with idiopathic FBS. Despite these limitations, our study highlights the characteristics of idiopathic FBS. In the future, we need to accumulate evidence from more cases to confirm our findings and discover therapeutic interventions in patients with idiopathic FBS.

## 5. Conclusion

We characterized the clinical and pain-related characteristics of idiopathic FBS in five patients without diabetes. Our findings indicate that these characteristics are largely consistent with those of postoperative FBS, thus supporting the notion that idiopathic FBS is a subtype of FBS. We emphasize the need to consider idiopathic FBS in the differential diagnosis of facial pain triggered at the beginning of a meal. The major clinical feature of idiopathic FBS pain that differentiates it from other types of facial pain is its reproduction with an acidic solution applied to the tongue.

## Figures and Tables

**Table 1:** Characteristics of patients with idiopathic first bite syndrome.

Patient no.	Age (years)	Sex	Duration	Initial diagnosis at the first consultation	Previous therapy
1	13	Female	2.5 months	TMD	Splint
2	44	Female	10 years	TMD	Splint
3	52	Female	5 years	Trigeminal neuralgia	Microvascular decompression, medication
4	53	Female	1.5 months	TMD, parotitis	No therapy
5	61	Male	3 years	TMD	Splint + laser therapy

TMD, temporomandibular disorder.

**Table 2:** Clinical findings in patients with idiopathic first bite syndrome.

Patient no.	Affected side	Parotid tenderness	Trigger zone	Triggering factor	Autonomic symptoms
1	Bilateral	Presence	Posterior of tongue	Taste stimuli	Orthostatic disturbance
2	Bilateral	Absence	Posterior of tongue	Taste stimuli	—
3	Left	Absence	Posterior of tongue	Taste stimuli	Miosis of the left eye, decreased sweating of the face
4	Left	Absence	Posterior of tongue	Taste stimuli	—
5	Right	Presence	Posterior of tongue	Taste stimuli	Miosis and ptosis of the right eye

**Table 3:** Imaging findings in patients with idiopathic first bite syndrome.

Patient no.	MRI of the TMJ	Dynamic salivary gland scintigraphy of the involved parotid glands		
		Time-activity curves	Accumulation	Rate of excretion (%)
1	Normal	—	—	—
2	Anterior disk displacement with reduction	Normal pattern	Good	Right: 96.7; left: 100
3	Anterior disk displacement with reduction	Normal pattern	Good	69.1
4	Normal	Normal pattern	Good	87.8
5	Normal	Normal pattern	Good	88.8

MRI, magnetic resonance imaging; TMJ, temporomandibular joint.

**Table 4:** Pain-related characteristics in patients with idiopathic first bite syndrome.

Patient no.	Pain intensity (NRS: 0–10)	Pain quality	Pain duration	Degree of interference with eating (NRS: 0–10)
1	10	Sharp	A few seconds	10
2	8	Sharp, electric	10 seconds	8
3	9	Sharp, tight	10–20 seconds	9
4	3	Sharp	A few seconds	5
5	10	Sharp, tight	10 seconds	10

NRS, numerical rating scale.

## Data Availability

The data used to support the findings of this study are included within the article.
